# Efficacy of sclerotherapy with pingyangmycin and polidocanol for venous malformations

**DOI:** 10.3389/fsurg.2025.1565333

**Published:** 2025-05-13

**Authors:** Guanghui He, Liping Zhang, Lei Guo, Aiqiang Han

**Affiliations:** ^1^ Department of Interventional Department, Weifang No. 2 People’s Hospital, Weifang, China; ^2^ Department of Vascular Anomalies and Interventional Radiology, Children’s Hospital Affiliated to Shandong University, Jinan, China; ^3^ Department of Gerontology, Weifang No. 2 People’s Hospital, Weifang, China

**Keywords:** sclerotherapy, pingyangmycin, polidocanol, venous malformation, treatment

## Abstract

**Objective:**

To investigate the treatment outcomes of sclerotherapy with pingyangmycin and polidocanol for venous malformations.

**Methods:**

This retrospective study included patients with venous malformations who underwent sclerotherapy between January 2022 and June 2024. Patient demographics, symptoms, treatment information were gathered from the electronic medical records, and imaging data from the hospital's picture archiving and communication system were reviewed.

**Results:**

A total of 29 patients, composed of 12 males and 17 females, with venous malformations, who underwent 66 procedures during the study period, were analyzed. The lesions were located in the lower limbs in 13 cases, in the upper limbs in 9, in the head and neck in 5, and in the trunk in 2. Twenty-four patients presented with pain, with a median visual analog scale (VAS) score of 4 and the correlation with age was statistically significant. After sclerotherapy, the median VAS score declined to 1, and there was a statistical difference in the overall median VAS score between pre- and post-treatment. According to the evaluation criteria, the treatment response was evaluated as grade 4 in 4 cases, as grade 3 in 17 cases, and as grade 2 in 8 cases; none of the treatment responses was evaluated as grade 1. None of the patients experienced serious adverse reactions.

**Conclusion:**

Sclerotherapy with pingyangmycin and polidocanol is a treatment modality for relieving the clinical symptoms of venous malformations, with good efficacy and few potential risks.

## 1 Introduction

Venous malformations (VMs) are the most common type of low-flow vascular malformations, accounting for almost 75% (1), with 1–2 cases found in more than 10,000 newborns (2). Although their natural history is usually benign, they can cause local and systemic complications resulting in significant morbidity, pain, and disability (3). Currently, treatment modalities for venous malformations include conservative observation, interventional sclerotherapy, surgical excision, laser treatment, and sirolimus, with sclerotherapy having emerged as the first-line treatment. Various sclerosing agents have been used in the management of VMs. However, reports on the combined application of different sclerosing agents are limited. The purpose of this article was to present and discuss the operational methods and clinical experience of combined sclerotherapy for venous malformations with pingyangmycin and polydocanol in a real-world study in China.

## 2 Patients and methods

This retrospective review specifically focused on patients with VMs who underwent sclerotherapy at our hospital between January 2022 and June 2024. Approval for the study was obtained from the hospital's ethics committee to ensure compliance with ethical guidelines. Electronic medical records were used to collect information on patient demographics, symptoms, complications, treatment modalities, and overall prognosis. Imaging data from the hospital's picture archiving and communication system were also reviewed, including preprocedural imaging, sclerotherapy techniques, and post-treatment follow-up imaging. Patients who did not meet the compliance criteria or had incomplete data were excluded from the analysis.

**Figure 1:**
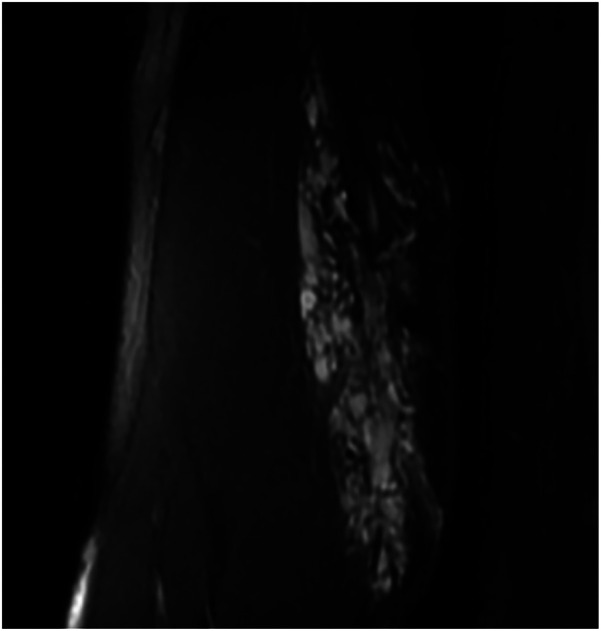
Male child, right upper limb mass with pain, VAS score: 4, large lesion visible on MR.

VMs were diagnosed based on clinical symptoms, ultrasonography (US), and magnetic resonance imaging (MRI) (Figure 1), following the International Society for the Study of Vascular Anomalies Classification 2018 (4). In addition, all patients were required to undergo electrocardiography, as well as the necessary laboratory tests, such as blood counts, coagulation function indices, and biochemistry, before being treated with sclerotherapy. And 1 case presented with localized intravascular coagulopathy (LIC) and received periprocedural anticoagulation therapy prior to sclerotherapy.

All treatments were performed by experienced interventional radiologists using digital subtraction angiography (DSA) and US guidance under general anesthesia or conscious sedation. The sclerosing agents used in this study were polidocanol foam and pingyangmycin, used alone or in combination with other agents. Polidocanol foam was prepared using 3% polidocanol and room air at a ratio of 1:4, according to the Tessari method (5, 6). Pingyangmycin was used as a liquid solution, composed of 8 mg of pingyangmycin, 1 ml (2 mg) of dexamethasone, and 3 ml of iodixanol. The cumulative dosage of the sclerosants never exceeded 2 mg/kg for 3% polidocanol and 0.5 mg/kg for pingyangmycin, with a maximum limit of 8 mg per session for pingyangmycin.

The venous malformation was punctured with a 4.5-scalp needle under US guidance. A syringe was withdrawn to visualize the return of blood within the venous space and confirm the intraluminal position of the tip of the needle. Venography was performed by injecting iodine contrast through the scalp needle to confirm the morphological configuration of the malformed venous mass and the anatomy of the draining veins. For high-flow lesions, polidocanol foam was injected into the VM under DSA contrast monitoring until the iodine contrast of the target lesion was replaced with foam sclerotherapy. For low-flow venous malformations, sclerotherapy was performed under DSA fluoroscopy with pingyangmycin.

**Figure 2:**
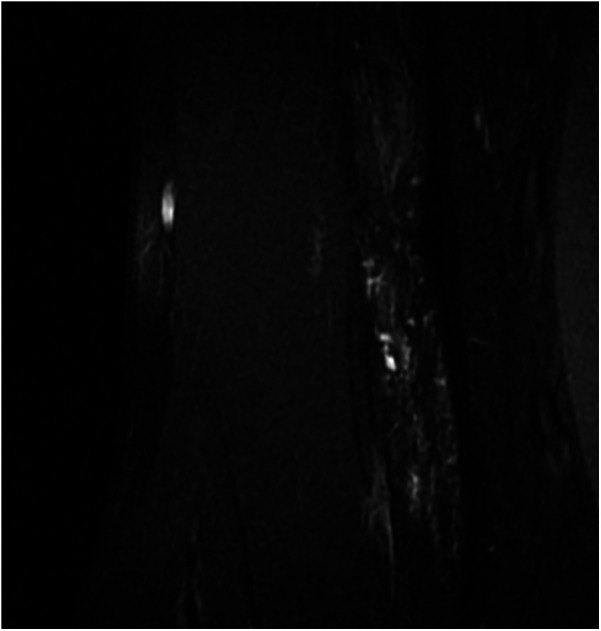
Pain relieved after 2 interventions, VAS score: 0, only a small residual lesion visible on MR. VAS, visual analog scale.

The evaluation of the treatment response in this study was accomplished primarily through clinical and radiological improvements, including volume reduction on MRI or US. Grading according to the degree of improvement in clinical symptoms was as follows: grade 1 (unchanged) with no improvement in symptoms and signs; grade 2 (slightly improved) with slight relief of pain, numbness, or swelling, or slight improvement in dysfunction or skin pigmentation; grade 3 (moderately improved) with reduction of pain, numbness, or swelling to a tolerable level, recovery of function to a level that can satisfy daily life, or significantly improved skin pigmentation; and grade 4 (significantly improved) with no pain, numbness, swelling, dysfunction, and skin pigmentation (7). Grading according to the US criteria was as follows: grade 1, with a hypoechoic lesion, volume reduction <40%, and abnormal blood flow signals; grade 2, with a moderately echogenic lesion, volume reduction ≥40% and <60%, and disappearance of abnormal blood flow signals; grade 3, with a hyperechoic lesion, volume reduction ≥60% and <80%, and disappearance of abnormal blood flow signals; and grade 4, with a hyperechoic lesion, volume reduction ≥80%, and complete disappearance of abnormal blood flow signals (8). Grading according to MRI (Figure 2) was as follows: grade 1, with increase in lesion volume by ≤10% or decrease in lesion volume by <10%; grade 2, with decrease in lesion volume by ≥10% but <25%; grade 3, with decrease in lesion volume by ≥25% but <50%; and grade 4, with decrease in lesion volume by ≥50% (9). The severity of adverse events (AEs) was determined according to the Society of Interventional Radiology reporting standards (10).

In the analysis of the collected data, descriptive statistics were employed to effectively summarize and characterize the findings. To evaluate the normality of the data distribution, the Shapiro–Wilk test was conducted. Depending on the outcome of this assessment, the data are reported either as the mean along with the standard deviation (SD) and the range, or, if the distribution did not meet the criteria for normality, as medians with the interquartile range (IQR) and the range. The data analysis was performed using SPSS software, specifically version 25.0. For this study, the threshold for statistical significance was set at *α* = 0.05, indicating that a *P* < 0.05 would be considered statistically significant.

## 3 Results

A total of 29 patients with VMs treated with sclerotherapy were included in this study, composed of 12 males and 17 females, with average age at first treatment was 9.89 ± 5.15 years (range: 0.92–21 years). The lesions were located in the lower limbs in 13 cases, in the upper limbs in 9, in the head and neck in 5, and in the trunk in 2. Nineteen of the included patients were examined for the first time, while the remaining 10 had undergone various courses of sclerotherapy, including one surgical resection. In addition, 8 patients had deep lesions with normal skin color on the body surface, while 21 patients had cyanotic lesions visible on the skin surface, of which 13 had only superficial lesions and 8 had mixed superficial and deep lesions. Patient details are shown in Table 1.

**Table 1:** Details of the medical records with VMs.

Variable	*n* = 29 patients
Sex	
Female	17 (58.62%)
Male	12 (41.38%)
Age (years)	9.89 ± 5.15
Location	
Head neck	5 (17.24%)
Lower limb	13 (44.83%)
Upper limb	9 (31.03%)
Trunk	2 (6.70%)
Postural testing	
+	21 (72.41%)
−	8 (27.59%)
Pain	24 (61.54%)

Twenty-one of the 29 patients presented with significant postural testing results, whereas the remaining 8 did not. Twenty-four patients presented with pain, with median visual analog scale (VAS) score was 4 (2, range: 1–7), mainly intermittent after activity and on pressure. And the association between VAS and age was statistically significant (*r* = 0.431, *p* = 0.02).

A total of 66 procedures were performed in 29 patients (Table 2), with median number of treatments was 2 (0) procedures (range: 1–6 procedures). Twenty-seven patients experienced relief from clinical symptoms, and in the 24 patients presenting with pain, a decrease in VAS scores of varying degrees was observed, with median VAS score was 1(2, range: 0–3). There was a statistical difference in the overall median VAS score between pre- and post-treatment (*Z* = −4.321, *p* < 0.001). According to the evaluation criteria, the treatment response was evaluated as grade 4 in 4 cases, as grade 3 in 17 cases, and as grade 2 in 8 cases. None of the treatment responses was evaluated as grade 1. In addition, none of the patients experienced serious adverse reactions. Correlation analysis of patients' prognosis with age, gender, position, postural testing, VAS, and NO of sclerotherapy showed a statistically significant correlation between prognosis and NO of sclerotherapy (*r* = −0.424, *p* = 0.022), and a statistically insignificant correlation with the other factors, but multifactorial regression analyses were performed to show a statistically insignificant correlation between prognosis and none of the above factors included.

**Table 2:** Details of sclerotherapy for VMs.

Total NO. sclerotherapy	66
Median NO. sclerotherapy	2 (range: 1–6)
Previous treatment history	
No	19 (65.52%)
Yes	10 (34.48%)
Median VAS score	*p* < 0.001
Preoperative	4 (range: 1–7)
Postoperative	1 (range: 0–3)
Prognosis	
2	8 (27.59%)
3	17 (58.62%)
4	4 (13.79%)

## 4 Discussion

VMs are congenital disorders that occur at different ages, including later in life (11). And within children, it can also be confused with infantile hemangiomas, which often regress without treatment, thus avoiding the endorsement of overtreatment. VMs can exist alone or in combination with other types of vascular malformations (12). Patients with VMs often experience symptoms such as swelling, pain, and a disfiguring appearance, which seriously affects their quality of life (11). In our study, pain was present in 24 of the 29 patients. Tissue function was limited to varying degrees in 8 patients and was more pronounced in older children, and there was a statistically significant correlation between VAS and age, consistent with previous reports stating that age is an important factor for pain incidence due to VMs (13).

Currently, the common treatment for venous malformations is sclerotherapy, using sclerosing agents such as ethanol, bleomycin, pingyangmycin, and polidocanol. Among these, ethanol showed the best effect on VMs but had the highest incidence of complications (1), which was not investigated in this study. Polidocanol has been successfully used in the sclerotherapy of VMs with satisfactory clinical results (14, 15). Pingyangmycin, also known as bleomycin A5, belongs to the bleomycin family, another commonly used sclerosant for VMs (16, 17). It is more effective and safer than traditional bleomycin (bleomycin A2 and bleomycin B2), and consequently, is more prevalently used clinically in China (1). In this study, both polidocanol and pingyangmycin were used, with pingyangmycin solution administered for tiny malformed vessels with slow flow rates and polidocanol foam for malformed lesions with sufficient diameters, volumes, and flow rates. In our opinion, this regimen ensures therapeutic efficacy while minimizing the occurrence of complications. The results of this study showed significantly fewer complications than previously reported (18, 19).

In addition, the use of imaging tests for the diagnosis and treatment of VMs should be paid attention to. US has emerged as an appropriate and reliable diagnostic tool for several pediatric musculoskeletal pathologies (20). VMs are characterized as hypoechoic or heterogeneous, and are seen as compressible lesions on US (21). As a screening tool, pre-treatment US can differentiate venous malformations from other vascular anomalies, such as infantile hemangiomas, lymphatic malformations, and arteriovenous malformations. Simultaneously, it can guide clinicians in puncturing the lesion intraoperatively, thereby reducing the risk of bleeding, extravascular injection, and accidental penetration of normal tissues/vessels. Fat-saturation T2-weighted MRI and T1 spin-echo sequences are the gold standards for pre-treatment evaluation of VMs (22). MRI can visualize the location of the lesion, its morphological features, and vital adjacent structures. It is also used to assess response to treatment and serves as the basis for subsequent treatment decisions, making it an integral part of most follow-up visits for VMs (23). Note that venous malformations, as a class of congenital diseases, are treated based on the principle of symptom control and improvement in the quality of life, rather than the complete elimination of the lesions. MRI also demonstrates residual lesions, which is perhaps one of the reasons for the low number of adverse reactions in this study. Providers may elect to proceed with observation in asymptomatic patients, given that waiting until after the onset of puberty does not increase the procedural load on patients (24). In this study, we did not find a correlation between age and treatment frequency (*r* = −0.044, *p* = 0.822); however, for patients with symptoms, timely treatment is recommended to alleviate the symptoms and to avoid comorbidities that may affect growth, development, and quality of life.

However, as a retrospective study, there may be a selection bias in this article and the small sample size reduces the statistical power. And the follow - up was short which as a limitation is crucial for assessing treatment sustainability. These aspects will guide future research.

## 5 Conclusions

As the disease naturally progresses, venous malformations can produce symptoms such as pain and dysfunction. Sclerotherapy, the first-line treatment for venous malformations, is efficacious in reducing the size of the lesion as well as relieving the accompanying pain symptoms. And sclerotherapy with pingyangmycin and polydocanol is safe and simple to perform. In addition to clinical experience, the use of well-established imaging tools can reduce the incidence of adverse reactions.

## Data Availability

The original contributions presented in the study are included in the article/Supplementary Material, further inquiries can be directed to the corresponding author.
